# CD4 count at presentation for HIV care in the United States and Canada: Are those over 50 years more likely to have a delayed presentation?

**DOI:** 10.1186/1742-6405-7-45

**Published:** 2010-12-15

**Authors:** Keri N Althoff, Kelly A Gebo, Stephen J Gange, Marina B Klein, John T Brooks, Robert S Hogg, Ronald J Bosch, Michael A Horberg, Michael S Saag, Mari M Kitahata, Joseph J Eron, Sonia Napravnik, Sean B Rourke, M John Gill, Benigno Rodriguez, Timothy R Sterling, Steven G Deeks, Jeffrey N Martin, Lisa P Jacobson, Gregory D Kirk, Ann C Collier, Constance A Benson, Michael J Silverberg, James J Goedert, Rosemary G McKaig, Jennifer Thorne, Anita Rachlis, Richard D Moore, Amy C Justice

**Affiliations:** 1 Department of Epidemiology, Johns Hopkins Bloomberg School of Public Health, 615 N Wolfe St., Baltimore, MD, 21205, USA; 2 Department of Medicine, Johns Hopkins University School of Medicine, 1830 E Monument St., Baltimore, MD, 21287, USA; 3 Department of Medicine, McGill University, 3650 Saint Urbain, Montreal, QC, H2X 2P4, Canada; 4 Division of HIV/AIDS Prevention, Centers for Disease Control and Prevention, 1600 Clifton Rd, Atlanta, GA, 30333, USA; 5 British Columbia Centre for Excellence and HIV/AIDS and Simon Fraser University, 608 - 1081 Burrard Street, Vancouver, BC, V6Z 1Y6, Canada; 6 Department of Biostatistics, Harvard University, 651 Huntington Ave, Boston, MA, 02115, USA; 7 Division of Research, Kaiser Permanente Northern California, 2000 Broadway, Oakland, CA, 94612, USA; 8 Department of Medicine, University of Alabama at Birmingham, 845 19th St South, Birmingham, AL, 35294, USA; 9 Department of Medicine, University of Washington, 325 Ninth Ave, Seattle, WA, 98104, USA; 10 Department of Medicine, University of North Carolina at Chapel Hill, Mason Farm Rd, 2101 Bioinformatics Bldg, Chapel Hill, NC, 27599, USA; 11 Departments of Psychiatry and Neuroscience, University of Toronto, 30 Bon St, Toronto, ON, M5B 1W8, Canada; 12 South Alberta HIV Clinic, University of Calgary, #3223, 1213 - 4th St SW, Calgary, AL, T2R 0X7, Canada; 13 Department of Medicine, Case Western Reserve University, 11000 Euclid Ave, Cleveland, OH, 44106, USA; 14 Department of Medicine, Vanderbilt University, 1161 21st Ave, Nashville, TN, 37232, USA; 15 Department of Medicine, University of California San Francisco, 50 Beale St, San Francisco, CA, 94105, USA; 16 Department of Epidemiology and Biostatistics, University of California San Francisco, 185 Berry St, San Francisco, CA, 94107, USA; 17 Department of Medicine, University of California San Diego, 220 Dickinson St, San Diego, CA, 92103, USA; 18 Division of Cancer Epidemiology & Genetics, National Cancer Institute, National Institutes of Health, 6120 Executive Boulevard, Bethesda, MD, 20892, USA; 19 Division of AIDS, National Institute of Allergy and Infectious Diseases, National Institutes of Health, 6700B Rockledge Dr., Bethesda, MD, 20892, USA; 20 Wilmer Eye Institute, Johns Hopkins University School of Medicine, 550 North Broadway, Baltimore, MD, 21205, USA; 21 Department of Medicine, University of Toronto, 2075 Bayview Ave, Toronto, ON, M4N 3M5, Canada; 22 Department of Medicine, Yale University School of Medicine and the VA Connecticut Healthcare System, 950 Campbell Ave, West Haven, CT, 06516, USA

## Abstract

We assessed CD4 count at initial presentation for HIV care among ≥50-year-olds from 1997-2007 in 13 US and Canadian clinical cohorts and compared to <50-year-olds. 44,491 HIV-infected individuals in the North American AIDS Cohort Collaboration on Research and Design (NA-ACCORD) were included in our study. Trends in mean CD4 count (measured as cells/mm^3^) and 95% confidence intervals ([,]) were determined using linear regression stratified by age category and adjusted for gender, race/ethnicity, HIV transmission risk and cohort. From 1997-2007, the proportion of individuals presenting for HIV care who were ≥50-years-old increased from 17% to 27% (p-value < 0.01). The median CD4 count among ≥50 year-olds was consistently lower than younger adults. The interaction of age group and calendar year was significant (p-value <0.01) with both age groups experiencing modest annual improvements over time (< 50-year-olds: 5 [4 , 6] cells/mm^3^; ≥50-year-olds: 7 [5 , 9] cells/mm^3^), after adjusting for sex, race/ethnicity, HIV transmission risk group and cohort; however, increases in the two groups were similar after 2000. A greater proportion of older individuals had an AIDS-defining diagnosis at, or within three months prior to, first presentation for HIV care compared to younger individuals (13% vs. 10%, respectively). Due to the increasing proportion, consistently lower CD4 counts, and more advanced HIV disease in adults ≥50-year-old at first presentation for HIV care, renewed HIV testing efforts are needed.

## Findings

We recently reported that the median CD4 count at first presentation for HIV care in the US and Canada increased from 256 (IQR: 96-455) to 317 (IQR: 135-517) from 1997 to 2007, yet remained below 350 cells/mm^3 ^- the current cut-off for initiating highly active antiretroviral therapy (HAART) [1,2]. Over the study period, there was an increase in the median age at first presentation for HIV care (from 40 to 43 years in 1997 to 2007, p < 0.01) [1]. According to the Centers for Disease Control and Prevention (CDC) 10% of the total incident HIV infections occurring in the US in 2006 were among adults ≥50-years-old [3]. Further, the prevalence of HIV infection in individuals ≥50 years of age is rapidly increasing [4,5], yet there is evidence that this older age group may not be as aware of HIV infection and the need for preventive measures and less likely to be tested and seek care early [6-9]. As this is the largest cohort collaboration of HIV-infected individuals in North America, we have conducted a new analysis that focuses on CD4 at first presentation for HIV care among patients ≥50-years-old.

We briefly describe study population and analytical methods; more details are provided in Althoff et al. [1].

All patients were enrollees in clinical care cohorts contributing to the North American Cohort Collaboration on Research and Design (NA-ACCORD) [10], a regional group of the International Epidemiological Databases to Evaluate AIDS (IeDEA) project. Each cohort's participation in NA-ACCORD was approved by the respective local institutional review boards. All 14 NA-ACCORD clinical cohorts agreed to participate in this study although one was excluded because their study population enrollment criteria restricted to those in later stages of HIV disease. These 13 clinical cohorts have clinical sites in 17 US states, Washington DC, and 3 Canadian provinces. Our primary focus was on HIV-infected adults who were ≥50 years of age and who first presented for clinical care between January 1997 and December 2007, as compared to individuals presenting at younger ages. First presentation for HIV clinical care was defined as the date (month and year) at which the first CD4 count was reported.

The first measured CD4 was our outcome of interest. The month and year in which the CD4 was measured were recorded. If there was more than one CD4 measurement in the first month at presentation for HIV care, we calculated the mean CD4 count for the month. Other information obtained at first presentation for care included self-reported year of birth, gender, race/ethnicity (as black, white, Latino and other/unknown) and HIV transmission risk group (male-to-male sex (MSM), injection drug use (IDU) including MSM/IDU, heterosexual contact and other/unknown).

Statistical comparisons of demographic and clinical characteristics across calendar dates were made using the Cochran-Armitage trend test for categorical variables or the Cuzick trend test for continuous variables. We determined the median absolute CD4 count and interquartile range (IQR) at first presentation for HIV clinical care annually from 1997 through 2007, by age group. Multivariate linear regression models were used to describe the annual trends in estimated mean CD4 count using a linear variable for year, stratified by age group and adjusting for cohort demographic and risk characteristics; 95% confidence intervals ([,]) were also estimated using these models. Sensitivity analyses were conducted by omitting participants from the Veterans Aging Cohort Study (VACS) and the HIV Research Network (HIVRN) as these two cohorts contribute ≈50% of the participants in the NA-ACCORD and the median age in the VACS was slightly older. Results with a two-sided p-value of <0.05 were considered statistically significant. Analyses were conducted using SAS, version 9.

After excluded individuals contributing data during the first year that the cohort contributed data to the NA-ACCORD to remove individuals who may have been previously in care, a total of 67,961 adults received HIV clinical care at one of the participating NA-ACCORD sites between 1997 and 2007 and had complete date and CD4 measurement information. Of these, 21,983 (32%) had a prior history of antiretroviral therapy or HIV-1 RNA results and 1,487 (2%) had an AIDS-defining diagnosis recorded more than 3 months prior to the first recorded CD4 count. These individuals were excluded as they were likely to have been previously in care. Our study population consisted of 44,491 HIV-infected individuals.

The proportions of individuals who were < and ≥50-years-old who first presented for HIV care each year are shown in Table 1; additional characteristics of the study population can be found in Althoff et al. [1]. From 1997-2007, the proportion of individuals presenting for HIV care who were aged ≥50 years increased from 17% to 27% (p-value < 0.01). The increase over time in median CD4 count at first presentation for care was similar in absolute magnitude in both age groups (67 cells/mm^3 ^and 63 cells/mm^3 ^from 1997 to 2007 among <50-year-olds and ≥50-year-olds, respectively). However, the ≥50-year-olds had a median CD4 count of 266 cells/mm^3^, compared to 336 cells/mm^3 ^among <50-year-olds, in 2007.

**Table 1:** Characteristics of N = 44,491 participating patients, by year at first presentation

	Total		1997		1998		1999		2000		2001		2002		2003		2004		2005		2006		2007		p-value^‡^
	N = 44,491		N = 4,479		N = 4,412		N = 4,857		N = 5,262		N = 4,258		N = 4,063		N = 3,688		N = 3,773		N = 3,486		N = 3,354		N = 2,859		
**Age (years)**																									
**18-< 50**	35,093	79%	3,698	83%	3,624	82%	3,953	81%	4,244	81%	3,344	79%	3,158	78%	2,855	77%	2,912	77%	2,709	78%	2,516	75%	2,080	73%	< 0.01
**≥50**	9,398	21%	781	17%	788	18%	904	19%	1,018	19%	914	21%	905	22%	833	23%	861	23%	777	22%	838	25%	779	27%	< 0.01
																									
**AIDS-defining illness**																									
**18-< 50**	3,390	10%	417	11%	385	11%	362	9%	370	9%	344	10%	331	10%	277	10%	270	9%	251	9%	208	8%	175	8%	< 0.01
**≥50**	1,242	13%	142	18%	127	16%	119	13%	121	12%	133	15%	119	13%	105	13%	105	12%	102	13%	95	11%	74	9%	< 0.01
																									
**CD4+ T-cell Count (cells/mm^3^)**																									
**18-< 50**																									
Median	298		269		277		275		284		293		313		296		312		323		333		336		< 0.01
IQR	112-493		100-467		96-481		99-464		104-494		100-494		124-501		114-504		127-499		134-500		141-512		152-522		
																									
**≥50**																									
Median	251		203		211		246		261		234		272		274		261		272		273		266		< 0.01
IQR	90-457		80-390		65-403		88-440		102-464		88-443		111-457		92-475		83-487		81-511		107-491		111-494		

^‡^P-values calculated using Cochran-Armitage test for categorical variables or Cuzick's test for continuous variables.

**Figure 1:**
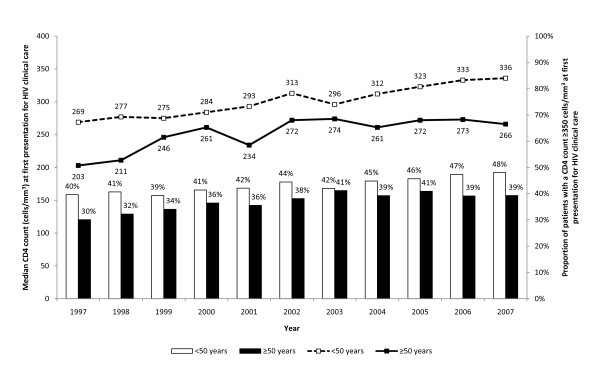
**Median CD4 count, and the proportion of individuals who have a CD4 count ≥350 cells/mm^3^, at first presentation for HIV clinical care**.

The median CD4 count was consistently lower in the ≥50-year-olds compared to the <50-year-olds from 1997 to 2007 (Figure 1). The proportion of individuals at first presentation for HIV care who had a CD4 count ≥350 cells/mm^3 ^was lower in the ≥50-year-olds compared to the <50-year-olds; this proportion increased over time for both age groups.

In the multivariate analyses, the estimated annual change in CD4 count from 1997 to 2007 was higher among ≥50-year-olds years (7 [5 , 9] cells/mm ^3^) compared to <50-year-olds (5 [4 , 6] cells/mm^3^) adjusting for sex, race and ethnicity, HIV transmission risk group and cohort. Findings were similar in sensitivity analyses. The interaction of age group and calendar year was statistically significant (p-value <0.01). After restriction to the years 2000-2007 in the ≥50-year-olds, the estimated annual change in CD4 count was 4 [1, 7] cells/mm^3^, similar to the change in the <50-year-olds from 1997-2007 (5 [4, 6] cells/mm^3^).

Overall, the proportion of individuals who had an AIDS-defining diagnosis recorded at, or 3 months prior to, the first CD4 measurement was highest among those aged ≥50 years (< 50-year-olds: 10%; ≥50-year-olds: 13%; p-value < 0.01); in sensitivity analyses, these proportions increased (< 50-year-olds: 12%; ≥50-year-olds: 18%; p-value < 0.01). The proportions who had an AIDS-defining diagnosis at first presentation for care decreased from 1997 to 2007 in both age groups (Table 1). Older individuals had a greater proportion with an AIDS-defining diagnosis in all years, however this disparity decreased over time (Table 1); in sensitivity analyses the decreases were of less magnitude (≥50-year-olds: 20% in 1997 to 15% in 2007, p-value < 0.01; <50-year-olds: 13% in 1997 to 12% in 2007, p-value < 0.01). Finally, among individuals who had an AIDS-defining diagnosis at first presentation for care, the proportion of older individuals who had ≥2 AIDS-defining diagnosis was similar to that of younger individuals (18% vs. 19%, p = 0.19).

Our study has three important findings: 1) the proportion of individuals at first presentation for care who are aged ≥50 years has increased over the past 11 years; 2) older individuals at first presentation of care consistently had a lower median CD4 count compared to younger individuals; and 3) a greater proportion of older individuals have an AIDS-defining diagnosis at, or within three months prior to, first presentation for HIV care compared to younger individuals.

The increase in the proportion of individuals who were ≥50 years at first presentation for care has implications for effective HIV management and survival for older infected individuals. Older individuals initiating HAART have a decreased immune response [11-18] and mortality increases with lower CD4 counts at HAART initiation [19]. In addition, older individuals at first presentation for care may have existing co-morbid conditions that may complicate HIV treatment decisions. From a public health perspective, a delay in presentation for treatment increases the risk for ongoing transmission [20-23]. These data suggest improved screening by health providers may help detect HIV infection earlier and at younger ages.

The estimated mean annual increase in CD4 count for individuals aged < and ≥50 years is small and likely of little clinical relevance as the within-patient variation in CD4 counts is ~25%. More importantly, the annual median CD4 count is still well below the CD4 recommended for initiation of HAART [24]. The proportion of individuals presenting with a CD4 ≥350 cell/smm^3 ^increased in all age groups, however, the proportion was approximately 10% lower among ≥50-year-olds. This suggests the potential for greater HIV treatment initiation guideline adherence if effective testing and treatment interventions target older individuals.

Finally, our data suggest older individuals are entering into care with advanced HIV disease. The CDC recently reported an increase in the proportion of ≥50-year-olds in the US who had a first HIV diagnosis within a year before AIDS diagnosis compared to 30-< 50-year-olds [25]; the Public Health Agency of Canada has noted the increase among ≥50 year-olds [26,27]. Data from New York City showed the proportion of new HIV diagnoses that are concurrent with an AIDS diagnoses increased with older age [28].

There are limitations to our study, including our lack of data regarding time since seroconversion. We chose to stratify the data using a cut-off of 50 years. Although there were more than enough individuals for additional stratification at younger ages, additional stratification at older ages was not possible.

While all age groups are experiencing modest improvements in CD4 count at presentation over time, older individuals have not "caught up." These data suggest that targeted renewed prevention and testing strategies are needed in all age groups, including those ≥50-years-old.

## Competing interests

Dr. Gebo reports receiving consulting fees from Tibotec and grant support from Johns Hopkins University Richard Ross Award, and Agency for Healthcare Research and Quality; Dr. Klein reports receiving consulting fees from GlaxoSmithKline, Abbott, Pfizer, and Merck, lecture fees from Abbott, Gilead, Tibotec, Bristol-Myers Squibb, and GlaxoSmithKline and research support from Canadian Institutes of Health Research/Fonds de la recherche en santé du Québec, Canadian HIV Trials Network, Ontario HIV Treatment Network, and Schering Plough Canada; Dr. Hogg reports receiving payment from a commercial entity that sponsored his study and grant support from Merck; Dr. Horberg reports receiving grant support from Pfizer, Merck, and Kaiser Permanente Community Benefits; Dr. Saag reports receiving consulting fees from Ardea Biosciences, Avexa, Boehringer-Ingelheim, Bristol-Myers Squibb, Gilead Sciences, GlaxoSmithKline, Merck, Monogram Biosciences, Pain Therapeutics, Pfizer, Progenics, Tibotec, Tobira Therapeutics, and Vicro and research support from Avexa, Achillion Pharmaceuticals, Boehringer-Ingelheim, Merck, Pfizer, Progenics, and Tibotec; Dr. Kitahata has served as a consultant to Gilead Sciences; Dr. Eron reports receiving consulting fees from Tibotec, Bristol-Myers Squibb, Merck, GlaxoSmithKline, Avexa, Tobira and Virco Labs, lecture fees from Roche, Bristol-Myers Squibb Virco Labs, and grant support from GlaxoSmithKline, Merck, and TaiMed; Dr. Gill reports receiving consulting fees from GlaxoSmithKline, Gilead, Abbott, Merck, Boehringer-Ingelheim, Thera, Tibotec, and Pfizer and grant support from GlaxoSmithKline, Abbott, Canadian Institutes of Health Research, Gilead, Tibotec, and Pfizer; Dr. Rodriguez reports receiving consulting fees from Gilead and Bristol-Myers Squibb, lecture fees from Bristol-Myers Squibb, and grant support from STERIS; Dr. Sterling reports receiving grant support from Pfizer; Dr. Deeks reports receiving grant support from Merck, Gilead, Bristol-Myers Squibb, and Pfizer; Dr. Collier reports receiving consulting fees from Merck, Pfizer, and GlaxoSmithKline, equity ownership/stock options in Bristol-Myers Squibb and Abbott, and grant support from Schering-Plough, Tibotec-Virco, Gilead, Boeringer-Ingelheim and Merck; Dr. Benson reports receiving consulting fees from GlaxoSmithKline, Pfizer, Merck, and Achillion, and grant support from Gilead; Dr. Silverberg reports receiving grant support from Pfizer and Merck; Dr. Rachlis reports receiving honoraria and research support from Bristol Myers Squibb, GlaxoSmithKline, Pfizer, Gilead, Tibotec, Schering-Plough, Merck, Theratechnologies, Abbott and the Ontario HIV Treatment Network; and Dr. Moore reports receiving consulting fees from Bristol-Myers Squibb and GlaxoSmithKline, lecture fees from Gilead, and grant support from Pfizer, Merck, Gilead, and Agency for Healthcare Research and Quality.

Drs. Althoff, Gange, Brooks, Rourke, Bosch, Martin, Jacobson, Kirk, Napravnik, Goedert, Buchacz, Thorne, McKaig and Justice declare they have no conflict of interest.

## Authors' contributions

KNA, KAG, SJG, RDM, and ACJ designed the study, interpreted the data, and drafted the manuscript; KNA also conducted the analysis. MBK, JTB, RSH, RJB, MAH made substantial contributions to the design of the study, interpretation of the data, and revised the manuscript critically for important intellectual content. MSS, MMK, JJE, SN, SBR, MJG, BR, TRS, SGD, JNM, LPJ, SDK, ACC, CAB, MJS, JJG, RGM, JT, AR oversee acquisition of data and revised the manuscript critically for important intellectual content. All authors approved the final manuscript.
